# A Heavy Issue: Changes in Body Size in London Before, During and After the Black Death

**DOI:** 10.1002/ajpa.70098

**Published:** 2025-07-17

**Authors:** Jessica Mongillo, Anthea Cerviero, Nicoletta Zedda, Natascia Rinaldo, Barbara Bramanti

**Affiliations:** ^1^ Department of Environmental and Prevention Sciences University of Ferrara Ferrara Italy; ^2^ Department of Neuroscience and Rehabilitation University of Ferrara Ferrara Italy

**Keywords:** bioarchaeology, black death, body mass, health changes, stature

## Abstract

**Objectives:**

The higher mortality rate of the Black Death compared to later epidemics has prompted bioarchaeologists to address the health status of past plague victims and whether this may have influenced the selectivity of the disease. A person's phenotype is the result of a complex interaction between genetic and environmental factors; body size, and in particular body mass and stature, are strongly influenced by external factors (such as economic conditions, famine, physiological stresses, etc.). In this study, we explored how variations in body mass and BMI may reflect changes in the population of London during the Black Death (1348–1350) and to understand the relationship between epidemic diseases and changes in body size in the past.

**Materials and Methods:**

For this purpose, stature, body mass, and BMI were reconstructed using osteologic data from the Wellcome Osteological Research Database (WORD) of the Museum of London from individuals who died before, during, and after the Black Death in medieval England.

**Results:**

We observed a significant decrease in weight and stature in the period of the Black Death and a slight increase, compared to pre‐Black Death data, after the plague epidemic. Values of BMI, conversely, remained more or less constant.

**Conclusions:**

The results further clarify the effects of social upheavals before the Black Death on the health of the individuals and reveal new insights into their health after the extreme devastation. Moreover, we confirmed that BM, as well as stature, can be used to assess health changes in the past.

**Summary**

- Body Mass can be used to assess health changes in the past.
- Black Death victims in London show a lower mean stature and weight due to previous famines.
- After the Black Death, health proxies' values improved considerably.

## 1 Introduction

The fourteenth century in Europe was marked by adverse climatic conditions which shortened food availability, with remarkable consequences for the socio‐economic stability of many countries. A series of famines, wars, and epidemics struck and weakened the European population in the first half of the century. These catastrophic events included the deadliest episode ever recorded, the so‐called Black Death, which lasted in Europe from 1348 until 1353, followed by numerous other episodes until the 19th century (Bramanti et al. 2018; Duncan and Scott 2005; Cohn 2008; Poos 1991; Wood et al. 2003; Wray 2005).

The rapid spread of this infectious disease and its high mortality rate that caused several changes in the social, economic, and political environment have prompted researchers to investigate the plague from different perspectives, including its bacteriological origin. In recent years, scientists have identified the bacterium *Yersinia pestis* in 14th‐century skeletal remains of putative plague victims, confirming its role as the causative agent (Haensch et al. 2010; Bos et al. 2011, 2016; Namouchi et al. 2018; Schuenemann et al. 2011; Spyrou et al. 2016, 2022).

Once the nature of the catastrophic events was ascertained and considering the higher mortality rate of the Black Death compared to subsequent outbreaks (Cohn 2008; Russell 1966), bioarchaeologists began to address the health status of plague victims of the past and whether this may have influenced the selectivity of the disease. In other words, the higher lethality of the Black Death could be owing to either the virulence of the *Y. pestis* strain or the previous poor health of the victims (DeWitte and Wood 2008; DeWitte 2014; DeWitte and Bekvalac 2010; DeWitte and Hughes‐Morey 2012). In the period immediately preceding the Black Death, in addition to malnutrition (Büntgen et al. 2011), the European population was affected by several other infectious diseases, such as smallpox, leprosy, tuberculosis (Barrett 1983; Behbehani 1983; Donoghue et al. 2005; Lee 2009; Manchester 1984; Sternbach 2003), which may have contributed to the worsening health status of many individuals, generating a discriminating factor for plague mortality (DeWitte 2009, 2010; DeWitte and Bekvalac 2010; DeWitte and Hughes‐Morey 2012; DeWitte and Wood 2008; DeWitte and Yaussy 2017; Godde et al. 2020; Zedda et al. 2022). In combination with poor health conditions, other endogenous characteristics may also have played a role in plague selectivity (DeWitte 2010; DeWitte and Bekvalac 2010); therefore, bioarchaeological studies on the Black Death and historical plagues in general have so far focused mainly on the analysis of frailty in relation to certain variables, such as sex, age, or stature (DeWitte 2009, 2010; DeWitte and Bekvalac 2010; DeWitte and Hughes‐Morey 2012; DeWitte and Wood 2008; DeWitte and Yaussy 2017, 2019; Godde et al. 2020; Yaussy et al. 2016; Yaussy and DeWitte 2019; Zedda et al. 2022; Guellil et al. 2021).

The phenotype of a person is the result of a complex interaction between genetic and environmental factors (e.g., McClearn 2004). Body size, and particularly weight and stature, are greatly affected by external factors that can distress growth, nutritional status, and health of an individual or population (Oreskovic et al. 2009), like social and financial circumstances, personal upheavals (i.e., stories of migrations, famine, physical and psychological traumas), conditions of physiological stress (Vilcins et al. 2018), as well as environmental changes, social status, activities carried out during life, and the spread of infectious diseases (Elliott et al. 2014; Houghton 1991; Ruff 2002; Sørensen et al. 2020; Wells 2012b, 2012a; Wells and Cortina‐Borja 2013). All these events can impact entire populations' daily activities and dietary habits, causing growth and body mass changes (Elliott et al. 2014; Ruff et al. 2005; Ruff 2002).

Body mass (BM) represents the total weight of an individual (without distinction between fat mass and lean mass) and is a key component of the individual's biological profile. In anthropological analyses, the reconstruction of BM can be used to analyze changes in body dimensions over time (Aiello and Wood 1994; Elliott et al. 2014; Ruff et al. 2005; Ruff 2002) but also to reconstruct the living conditions and lifestyles of a specific population (Lieberman et al. 2001; Lorkiewicz‐Muszyńska et al. 2013; Ruff et al. 2005; Squyres and Ruff 2015). Stature provides as well relevant information on the living conditions of past populations and is a crucial variable for reconstructing the size and body proportions of individuals from skeletal remains (Giannecchini and Moggi‐Cecchi 2008; Ruff et al. 2012). A combination of the two measures, BM and stature, gives the body mass index (BMI), which can also be used in both bioarchaeological and forensic contexts as a suitable index for implementing the biological profile of an individual (Mongillo et al. 2021). Although widely applied in clinical and epidemiological research, BMI remains poorly explored in bioarchaeology. When derived from osteological data, BMI estimates can provide valuable insights into weight status and associated comorbidities. Underweight conditions such as malnutrition, stunting, and wasting are known to be reflected in short stature, low bone mass, and increased porosity (Schug and Goldman 2014; Temple et al. 2014; Kueper et al. 2015), while overweight is associated with increased mechanical stress and skeletal conditions including osteoarthritis, DISH, gout, and calcaneal spurs (Jiang et al. 2012; Reyes et al. 2016; Wallace et al. 2017; Giuffra et al. 2010). Thus, as in living populations, BMI may serve as a proxy for nutritional and metabolic status, aiding population‐level reconstructions of health and disease.

While in previous studies, DeWitte and colleagues (DeWitte and Hughes‐Morey 2012; DeWitte 2017) investigated changes in stature during the Black Death, changes in BM have never been investigated; indeed, to the best of our knowledge, only one previous study, which used femoral head size as a proxy, considered BM as a marker of frailty in plague victims (Zedda et al. 2022).

In this study, we compared BM and estimated stature of individuals before, during, and after the Black Death, to evaluate the possible impact of the plague epidemic on changes in body size. Moreover, we specifically test the applicability of BMI to assess whether this indicator can effectively capture population‐level changes in weight conditions in the past. Data from the Wellcome Osteological Research Database (WORD) of the Museum of London are well suited for this type of analysis because they allow for diachronic comparisons between individuals and offer a unique opportunity to test our method in the context of significant population changes. From our analysis, we expect to find a general increase in the body size and health status of the population after the Black Death, since historical sources reported an improvement in the living standards after the plague with a redistribution of wealth and increased access to food (Dyer 2005; DeWitte 2015).

## 2 Materials and Methods

### 2.1 The Osteological Sample

Data examined in this study were obtained from the Wellcome Osteological Research Database (WORD) of the Museum of London (https://www.museumoflondon.org.uk/collections/other‐collection‐databases‐and‐libraries/centre‐human‐bioarchaeology/osteological‐database). This database includes demographic and anthropometric data of skeletonized individuals from several historical London cemeteries, made openly available free of charge by the Museum of London to anyone with a purpose of research. Five medieval cemeteries recorded in the WORD database were considered in this study, divided into three periods: pre‐Black Death (Period A, before the year 1348, specifically the period between 1050 and 1320), during Black Death (Period B, 1348–1350) and post‐Black Death (Period C, 1350–1550) (Table 1). Cemeteries without a precise chronological sequence were excluded from the study. The attribution of the individuals to one of the three periods was made according to the information available in the WORD database.

**TABLE 1:** Number of included individuals divided by sex and age‐at‐death classes for each cemetery and separated for periods: Pre‐ (Period A), During‐ (Period B) and Post‐Black Death (Period C).

Period	Cemetery	Chronological period	M	F	YA	MA	OA	Total
A	Guildhall yard (Periods 10, 11)	1050–1230	12	9	10	7	4	21
	Merton priory (Periods 3, 4)	1117–1300	41	8	14	26	9	49
	Spital square	1197–1320	27	18	19	19	7	45
	Total		80	35	43	52	20	115
B	East Smithfield Black death	1348–1350	127	57	103	67	14	184
	Total		127	57	103	67	14	184
C	Merton priory (Periods 7, 8)	1390–1550	9	0	2	7	0	9
	St. Mary Graces	1350–1538	70	30	44	43	16	100
	Total		79	30	46	50	16	109

*Note:* M: males; F: females; YA: 18 – 35 years of age; MA: 36–45 years of age: OA: > 46 years of age.

The overall initial sample consisted of a total of 1893 individuals. Of these, 413 individuals were excluded because their death time, indicated in the WORD Database, did not belong exactly to one of the three periods (A, B, C) previously specified. Of the remaining 1480 individuals, all sub‐adults, adults whose sex or age‐at‐death was indeterminate, and, lastly, all adults who did not have enough skeletal elements to determine stature and BM were not included in the final sample, which consists of 408 individuals. Because this study aims to propose a methodological approach, we did not include skeletal and dental pathologies associated with individual cases.

The final sample, divided by sex and age‐at‐death, is reported in Table 1.

#### 2.1.1 Pre‐Black Death Cemeteries (Period A)

The 121 Pre‐Black Death individuals came from three London cemeteries: Guildhall Yard, Merton Priory, and Spital Square.

##### 2.1.1.1 Guildhall Yard

Guildhall Yard was excavated between 1992 and 1997 (MoLAS‐Museum of London Archaeological Service). It was originally the parish churchyard of St Lawrence Jewry, dated between the 11th and 12th centuries, and is composed of high‐status parishioners (Bowsher 2007). Its timeline is divided into several periods. Only Period 10 (1050–1140 ce) and Period 11 (1140–1230 ce) were considered in this study.

##### 2.1.1.2 Merton Priory

The site of the Augustinian Priory of St Mary Merton was excavated in two separate archaeological surveys, from 1977 to 1983 by the DGLA (Department of Greater London Archaeology) and from 1986 to 1988 by MoLAS. A total of 738 burials with both single and multiple graves were found here, of both monks and lay people, including wealthy community members and servants (Miller and Saxby 2007). The graves are arranged over four main areas, divided into four time periods covering the years 1117–1538. In this study, individuals from Period 3 (1117–1222 ce) and Period 4 (1222–1300 ce) were considered, while Period 6 (1300–1390 ce) was excluded due to the difficulty in distinguishing individuals who died before the Black Death from those who died during and after it.

##### 2.1.1.3 Spital Square

The individuals buried here came from an Augustinian priory and the hospital of St Mary Spital and dated between 1197 and 1320. Excavations were carried out by the Museum of London between 1985 and 1989.

Archaeologically, four distinct areas were distinguished: area SSQ88 (1197–1235 ce) with individuals from the priory (1 individual included in this study); areas NRT85 and NRF88 (1235–1280 ce; respectively 20 and 18 individuals), with individuals from the hospital cemetery; and area SPQ88 (1280–1320 ce) with individuals from the first phase of the infirmary chapel (6 individuals included).

#### 2.1.2 Black Death Cemeteries (Period B)

The only cemetery considered here is the East Smithfield Black Death cemetery, with a very significant record of plague victims in London. It was excavated by the Museum of London from 1986 to 1988. In the WORD database are collected data on 636 individuals, from whom 188 were selected for this study.

The East Smithfield cemetery was founded in 1348 (Hawkins 1990; Grainger et al. 2008) to cope with the daily high mortality due to plague. Its structure demonstrates the emergency character of the site: in addition to single burials, three mass burial trenches were discovered which, from the study of the stratigraphy, appear to have been generated in a single phase (DeWitte and Hughes‐Morey 2012). Significantly for our study, there is no evidence of its use after 1350 (Grainger et al. 2008). In 1990, Hawkins suggested that Black Death victims were buried exclusively in plague cemeteries once established. Archeogenomic work supports this evidence (Bos et al. 2011).

#### 2.1.3 Post‐Black Death Cemeteries (Period C)

The post‐Black Death sample mostly consists of individuals from the St Mary Graces cemetery. In addition, we considered 9 individuals from the later phases of the Merton Priory, specifically eight individuals from Period 7 (1390–1538 ce) and one from Period 8 (1538–1550 ce).

The St Mary Graces cemetery, excavated from 1986 to 1988, was associated with the Cistercian abbey with the same name, which was founded in 1350 (just after the Black Death) and used until the Reformation of 1538 (Grainger et al. 1988, 2008). Individuals from this cemetery represent various social classes, as well as monks (Grainger and Phillpotts 2011). In this analysis, we included 20 individuals who died around 1400, 64 individuals from 1350, and 16 individuals from 1353. Among the individuals listed as dating between 1350 and 1400, two in particular appear to have been buried as a result of a plague outbreak after the Black Death, suggesting that they died from this disease (Gilchrist and Sloane 2006). However, given the purpose of this study, we decided to include them in the analysis because they belong to a period after the Black Death, which is our point of reference.

### 2.2 Sex and Age‐at‐Death Estimation

Data on sex and estimated age at death of the individuals considered in this study were extracted from WORD. To obtain this information from the bone remains, the Centre for Human Bioarchaeology used different methods (Powers 2012).

Sex was determined in adults by macroscopic observation of 14 sexually dimorphic features of the skull, jaw, and pelvis, following classical anthropological methods (Brothwell 1981; Bass 1987; Ferembach et al. 1980; Phenice 1969). Each observed element was assigned a score from 1 to 5, where 1 corresponds to ‘male’ and 5 to ‘female’; when no diagnostic elements were observable, sex code 9 was assigned.

Age‐at‐death was determined using a combination of methods: morphology of the pubic symphysis (Brooks and Suchey 1990; Buikstra and Ubelaker 1994), morphology of the auricular surface (Brooks and Suchey 1990; Buikstra and Ubelaker 1994; Lovejoy et al. 1985), morphology of the sternal ribs (Işcan et al. 1984, 1985) and grade of dental wear (Brothwell 1981).

In the present study, the age classes indicated in the WORD database were converted to the Ubelaker age‐at‐death classes: Young Adult (20–35; YA), Middle Adult (35–50; MA), Old Adult (50+; OA) (Buikstra and Ubelaker 1994).

### 2.3 Stature Estimation

We applied the formulas developed by Trotter and Gleser (1951, 1952, 1958, 1977) to estimate the stature of each individual of each period, using the maximum lengths of long bones (femur, humerus, radius, ulna, fibula) that are recorded in the WORD database. For all individuals older than 30 years, the measure was adjusted for age‐at‐death, as indicated by the authors of the formulas. In the case of individuals for whom only the maximal tibial length measure was available, we used Pearson's formulas (Pearson 1899). If present, left‐sided bones were preferred. If different lengths of long bones were present for an individual, stature was calculated from the measurements of each left bone and then the mean stature was calculated.

### 2.4 BM and BMI Estimation

BM estimation was performed according to the method of Trinkaus and Ruff (2012) based on vertical femoral head diameter (FHB). This method makes use of three different formulas (Ruff et al. 1991; Grine et al. 1995; McHenry 1992), chosen according to the different sizes of the FHB (McHenry 1992, if FHB < 38 mm; Ruff et al. 1991, McHenry 1992; Grine et al. 1995, if FHB between 38 and 47 mm; and Ruff et al. 1991; Grine et al. 1995, if FHB > 47 mm). To increase the number of individuals that could be considered, other formulas based on the measurements of the latero‐medial width of the tibial plateau (TPLM; Keisu et al. 2019) and the femoral bi‐epicondylar breadth (FBEB; Keisu et al. 2019) were used when FHB was not available. The formulas used for the evaluation of the BM in this study are reported in Table 2.

**TABLE 2:** Formulas used for the reconstruction of BM.

Skeletal element	Authors	Formulas
FHB (mm)	Ruff et al. 1991	♂BM = 2.741 × FHB—54.9 ♀BM = 2.426 × FHB—35.1
	McHenry 1992	♂ + ♀BM = 2.239 × FHB—39.9
	Grine et al. 1995	♂ + ♀BM = 2.268 × FHB—36.5
TPLM (mm)	Ruff et al. 2018	♂ + ♀BM = 1.623 × TPML—52.7
	Keisu et al. 2019	♂BM = 1.25 × TPML—22.75 ♀BM = 1.20 × TPML—23.02
FBEB (mm)	Keisu et al. 2019	♂BM = 1.07 × FBEB—15.88 ♀BM = 1.09 × FBEB—21.42

Abbreviations: FBEB, femoral bicondylar breadth; FHB, femoral head breadth; TPML, mediolateral measurements of tibial plateau.

Body mass index (BMI) was calculated using the estimated BM and stature through the formula BM (in kg)/stature^2^ (in meters squared). Following the World Health Organization (WHO) classification, weight status was classified according to cut‐offs: BMI values below 18.5 indicate underweight; BMIs between 18.5 and 24.9 indicate normal weight, whereas overweight is defined by a BMI of 25–29.9, and obesity by a BMI above 30 (James 2004).

### 2.5 Statistical Analysis

Statistical analyses were performed using the STATISTICA (StatSoft) software and MedCalc Statistical Software version 14.8.1 (MedCalc Software bvba, Ostend, Belgium). The assumption of normality was verified using the Kolmogorov–Smirnov test and the Shapiro–Wilk test. Mean and standard deviation (SD) were calculated for continuous variables (stature, BM, BMI), whereas relative and absolute frequencies for categorical variables (weight status) were reported. The Levene test and Bartlett test were used to verify the homogeneity of variances in different groups. Two‐way analysis of variance (ANOVA) was performed to determine whether stature, BM, and BMI (dependent continuous variables) differ significantly between sex (independent categorical factor), periods (independent categorical factor) and their interaction. Tukey post hoc test was performed to test differences within the three periods. The comparisons between weight status and sex and period, respectively, were computed using the Fisher–Freeman–Halton test. Three multiple linear regression models were performed to assess the association between stature, BM, and BMI—inserted as dependent continuous variables—and historical periods (categorical variables) as independent variables (crude models). Each model was then adjusted for sex (categorical variable) and age‐at‐death (categorical variable), added as confounding variables (adjusted models). The normality of the residuals and homoscedasticity were verified using the Levene test and the Shapiro–Wilk test. Variance Inflation Factor (VIF) and Generalized Variance Inflation Factor (GVIF) were performed to test the collinearity. All the assumptions for the Two‐way ANOVA test and the multiple linear regression models were met for all the considered variables (stature, BM and BMI). In all cases, values of *p* < 0.05 were considered to indicate statistical significance.

## 3 Results

**FIGURE 1:**
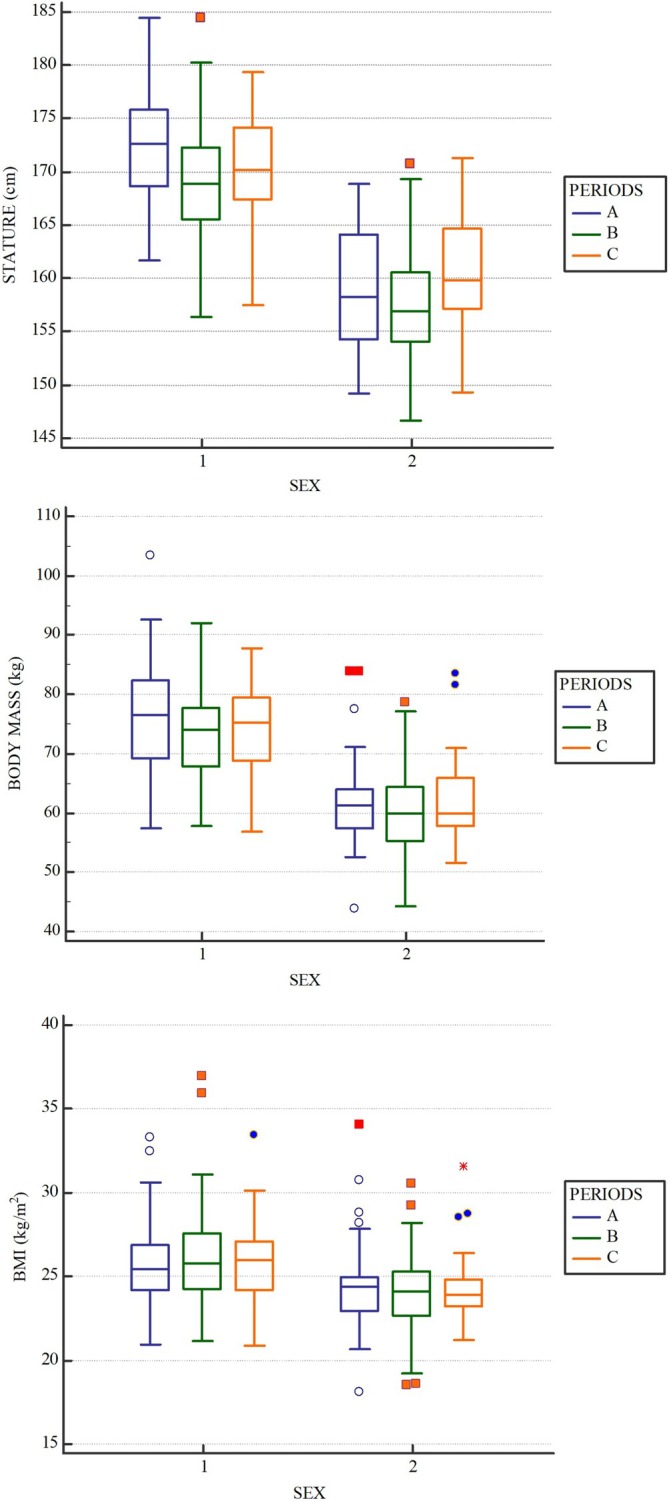
Boxplots illustrating the difference in the three periods separately by sex. (A) Differences in stature; (B) differences in BM; (C) differences in BMI. 1: Males; 2: Females.

Stature, BM, and BMI in the two sexes and in the three periods (Pre‐Black Death, Black Death and Post‐Black Death) are presented in Table 3 and Figure 1 (A, B, C). The results of the two‐way ANOVA show a highly significant difference between the two sexes for all variables considered. Males were taller and heavier than females and had a higher BMI, as expected.

**TABLE 3:** Results of two‐way analysis of variance (ANOVA) and Fisher–Freeman–Halton test showing the effects of sex and periods on stature, BM, and BMI.

Variables	Period A		Period B		Period C		DF sex	F sex	*p* value sex	DF periods	F periods	*p* value periods	DF interaction	F interaction	*p* value sex*period
	Males	Females	Males	Females	Males	Females									
	Mean (SD)	Mean (SD)	Mean (SD)	Mean (SD)	Mean (SD)	Mean (SD)									
	*N* = 80	*N* = 35	*N* = 127	*N* = 57	*N* = 79	*N* = 30									
Stature (cm)	172.2 (5.0)	159.0 (5.3)	168.8 (5.0)	157.6 (5.2)	170.4 (4.9)	160.8 (5.6)	1	396.7	< 0.001	2	9.4	< 0.001	2	2.9	0.055
BM (kg)	76.2 (8.0)	62.1 (8.0)	73.3 (7.0)	59.9 (7.1)	74.6 (6.9)	62.1 (7.1)	1	267.7	< 0.001	2	3.9	0.021	2	0.3	0.748
BMI (kg/m2)	25.6 (2.3)	24.6 (2.9)	26.0 (2.5)	24.1 (2.4)	25.8 (2.2)	24.3 (2.1)	1	29.8	< 0.001	2	0.0	0.999	2	0.8	0.425

Weight status	Period A		Period B		Period C		DF sex	*X* ^2^ sex	*p* value sex	DF periods	*X* ^2^ periods	*p* value periods
	*N* (%)	*N* (%)	*N* (%)	*N* (%)	*N* (%)	*N* (%)						
Underweight	0 (0.0)	1 (2.9)	0 (0.0)	0 (0.0)	0 (0.0)	0 (0.0)	3	56.345	< 0.001^a^	6	5.482	0.459^a^
Normal weight	35 (43.7)	29 (82.9)	46 (36.2)	40 (70.2)	26 (32.9)	24 (80.0)						
Overweight	42 (52.5)	5 (14.3)	76 (59.8)	16 (28.1)	52 (64.6)	5 (16.7)						
Obese	3 (3.7)	0 (0.0)	5 (3.9)	1 (1.8)	2 (2.5)	1 (3.3)						

^a^ Comparison performed via Fisher–Freeman–Halton test.

The mean BMI of the males fell into the overweight category in all periods, while the mean BMI of the women was almost overweight. When weight status categories were compared, males showed a significantly higher frequency of overweight individuals in all three periods, representing more than half of the total sample, in contrast to females, who were predominantly normal weight. The differences between periods were not statistically significant for both BMI and weight status categories, considering the total sample and each sex separately. Regarding stature and BM, the results show a significant difference between periods. The absence of serious violations of homoscedasticity and the sample size allow us to consider two‐way ANOVA results robust, even if the distribution of a few variables is not normal.

The post hoc test revealed that stature was significantly lower in the Black Death period (Period B) than in the periods before and after the Black Death (A and C), whereas no significant differences were found between the first and the last period. BM was only significantly different between periods A and B, with higher values in the first period. After the Black Death, we observe an increase in both stature and BM, with values not significantly different from the pre‐Black Death period. The increase in stature and weight after the Black Death, more marked for females, also translates into changes in BMI. Indeed, BMI values of males and females remain similar because there is a constant increase in both weight and stature (1.3 kg and 1.6 cm for males; 2.2 kg and 3.2 cm for females) after the Black Death.

Using multiple linear regression models, the association between the three measures (stature, BMI and BM) and the three periods (pre‐, during and post‐Black Death) was analyzed. The absence of multicollinearity between the independent variables (VIF/GVIF ≈1), along with the homoscedasticity of the residuals, confirms the validity of the model. The time period proved to be a good predictor of stature in both the crude and the adjusted models (Table 4, models 1 and 2), with an explained variance of respectively 3% and 52%. The results are similar when BM is inserted as the dependent variable. Indeed, in the second model, the explained variance increases to 42% in comparison to the first model (1%) (Table 5). Both stature and BM resulted significantly higher in period A and lower in period B in comparison to period C. As expected, regardless of the time period, males resulted significantly higher and heavier than females, and no significant differences were found comparing the age‐at‐death classes. Concerning BMI, the model was statistically significant only after adjusting for sex and age‐at‐death. However, the period was not significantly associated with BMI in any model (crude and adjusted). YA had a significantly lower BMI in comparison to OA, regardless of sex and period. The covariates of the adjusted model explained 9% of the variance (Table 6). Looking in detail at the distribution of individuals in the different age groups (Table S1), the trend of increasing stature for females and males after the Black Death is maintained for almost all three adult groups considered, except for YA males whose stature is similar in periods B and C. However, for BM, a decrease is noted for both males and females in the Black Death period, but the values are similar for the B and C periods for YA males, while a very slight decrease is shown for MA males. For the female OA age group, after the Black Death, there is a decline in BM measures.

**TABLE 4:** Multiple linear regression model showing the association between stature and plague periods (Model 1, crude model) and after adjusting for covariates (Model 2, adjusted model).

Stature	Model 1			Model 2		
	*β*	*t*	*p*	*β*	*t*	*p*
Periods						
A	0.111	2.051	0.041	0.124	3.263	0.001
B	−0.197	−3.634	0.000	−0.187	−4.830	0.000
C	ref	ref	ref	ref	ref	ref
Sex (male)	—	—	—	0.701	20.430	0.000
Age‐at‐death	—	—	—			
YA	—	—	—	0.033	0.943	0.346
MA	—	—	—	0.035	1.031	0.303
OA	—	—	—	ref	ref	ref
*P* value	0.001			0.000		
R2	0.032			0.529		
R2 adjusted	0.027			0.523		

**TABLE 5:** Multiple linear regression model showing the association between BM and plague periods (Model 1, crude model) and after adjusting for covariates (Model 2, adjusted model).

Body mass	Model 1			Model 2		
	*β*	*t*	*p*	*β*	*t*	*p*
Periods						
A	0.090	1.651	0.099	0.093	2.204	0.028
B	−0.135	−2.478	0.014	−0.111	−2.602	0.010
C	ref	ref	ref	ref	ref	ref
Sex (male)	—	—	—	0.642	16.962	0.000
Age‐at‐death	—	—	—			
YA	—	—	—	−0.045	−1.159	0.247
MA	—	—	—	−0.025	−0.664	0.507
OA	—	—	—	ref	ref	ref
*P* value	0.039			0.000		
R2	0.016			0.428		
R2 adjusted	0.011			0.421		

**TABLE 6:** Multiple linear regression model showing the association between BMI and plague periods (Model 1, crude model) and after adjusting for covariates (Model 2, adjusted model).

BMI	Model 1			Model 2		
	*β*	*t*	*p*	*β*	*t*	*p*
Periods						
A	−0.027	−0.484	0.628	−0.038	−0.725	0.469
B	−0.017	−0.313	0.755	0.049	0.912	0.362
C	ref	ref	ref	ref	ref	ref
Sex (male)	—	—	—	0.283	5.951	0.000
Age‐at‐death	—	—	—			
YA	—	—	—	−0.120	−2.484	0.013
MA	—	—	—	−0.056	−1.184	0.237
OA	—	—	—	ref	ref	ref
P value	0.883			0.000		
R2	0.001			0.097		
R2 adjusted	−0.004			0.086		

## 4 Discussion

The aim of the study was to examine whether variations in body size can serve as an indirect measure of changes in physical conditions over time in relation to a specific historical context, events, and circumstances. In particular, we considered the medieval cemeteries of London before, during, and after the Black Death, which provide a valuable framework for critically investigating this matter and a direct understanding of the relationship between epidemic diseases and changes in body size in the past.

The relationship between weight status and morbidity and mortality of infectious diseases in historical times remains an open issue in the scientific literature (Schneider 2022). Nutritional status and disease mutually influence each other; infectious disease, regardless of the agent responsible for the infection, can affect nutritional conditions through reduced appetite and loss of nutrients (Katona and Katona‐Apte 2008). Conversely, pre‐existing nutritional deficiencies, thus weight status, may increase the severity of some infections by impairing immune system functions and reducing antibody production (Schneider 2022; Bellagio Conferees 1983). Indeed, some studies have suggested that BM could be associated with infection risk rate and disease outcome (Yang et al. 2021; Dobner and Kaser 2018). Malnutrition in general increases the risk of developing more severe infections, especially in underweight children and adolescents, and obese adults (Dobner and Kaser 2018; Krawinkel 2012).

However, it is difficult to apply estimates of modern medicine to the past, as antibiotics and vaccinations have changed the contagion probability and severity of most diseases (Schneider 2022). Historical sources on past health practices and bioarchaeological analysis can help obtain information and draw a picture of the health status in the past.

This study represents the first attempt to analyze changes in BM and BMI, to assess weight status and infer the health conditions of the London population, in relation to the most severe 14th‐century plague outbreak, the Black Death. The London sample selected for the analysis is part of a larger collection curated by the London Museum, which includes human remains from different prehistoric and historical periods, each accompanied by information such as chronology, the context of discovery, and anthropological information, including osteometric measurements, recorded according to professional standards (Powers 2012). Such a large sample allowed us to understand the change in BM before, during, and after the Black Death.

As expected, the results confirm sexual dimorphism in BM and stature in all three periods. It is well known that sexual dimorphism in human body size begins from fetal life; moreover, boys enter puberty later than girls and continue the pubertal growth spurt by increasing leg size and length and total bone mass (Wells and Cole 2002). This prolonged growth process also stimulates periosteal apposition, leading to increased bone diameters, unlike in females, where estrogens inhibit the process. Delays in puberty may interfere with sex differences (Wells 2007; Seeman 2001).

Sexual dimorphism in stature in the London population has already been reported in previous studies (DeWitte 2018; DeWitte and Hughes‐Morey 2012; Brennan and DeWitte 2022). Among the three periods considered, sexual dimorphism in BM, stature, and thus BMI remained more or less constant, with more marked differences in period A and less in period C.

However, we observed that in all three periods, there is a notable frequency of overweight individuals when considering BMI. This condition has also been reported in other attempts to estimate body mass using BMI, as in the study of Siegmund and Papageorgopoulou (2011) in medieval Switzerland. However, as Ruff (2000) and Weiss (2007) have demonstrated, high BMI values in premodern contexts do not necessarily correspond to modern patterns of overnutrition. Increased physical activity likely contributed to increased muscle mass, affecting BM without indicating poor health. We know that in the period right before the Black Death, two main famines struck England: The Great Famine in 1315 and 1317, followed by the Great Bovine Pestilence in 1319–1320 (DeWitte and Slavin 2013; Stothers 2000). As a result of these circumstances, a significant proportion of the English population experienced declining living standards and a rise in social injustice. These conditions not only led to a general decline in health status shortly before the Black Death but also caused an important migration into the city of London of sick and disease‐prone immigrants from the poorer countryside (DeWitte 2015, 2017; Campbell 2016; Stothers 2000). Malnutrition, starvation, and stress during fetal life and the early years of childhood can stunt growth, have a long‐lasting or permanent adverse impact on immune function, and consequently raise the risk of infectious disease‐related mortality in adults (DeWitte 2017; Moore et al. 1999; Moore 2016; Godde et al. 2020). Given that stature is genetically defined and influenced by environmental factors, it is known that children who have suffered episodes of infection could have a shorter stature in adulthood (Rani et al. 2021; Zedda et al. 2021). On the other hand, weight undergoes many fluctuations over a lifetime, sometimes very rapid ones, which are difficult to predict from the skeleton. We also know that BM estimation methods based on joint size reflect weight at the time the bone is setting (18–20 years of age for the femoral head) or when peak weight has been reached (30–40 years of age for knee joints) (Trinkaus and Ruff 2012; Squyres and Ruff 2015; Elliott et al. 2014; Ruff et al. 1991).

Therefore, it is conceivable that the significant decrease in weight and stature during the period of the plague observed in the results of our work could be explained by the fact that some individuals who died of the plague had suffered the effects of pestilences and famine during childhood or the fetal stage. The victims of the plague might mirror the status of the population of the time, with a lower mean stature and weight due to the previous famines: but they also may represent the frailest individuals who were selected by the plague because they had suffered stress in earlier stages of life and different studies have reported an increased susceptibility to dying of plague for frailer individuals. Nevertheless, in plague pits a higher than expected presence of individuals with a lower level of frailty (i.e., healthier) was observed as well (Zedda et al. 2022; DeWitte and Wood 2008; DeWitte and Hughes‐Morey 2012).

In this study, we could observe in particular the lower weight of the male cohort of young adults (20–35 years), who had experienced periods of famine and pestilence when they still were children, resulting in a compromised health status that might have made them more susceptible to dying of plague. Even in multiple linear regression models, the three measures (stature, BMI and BM) and time period displayed a significant association, suggesting that lower stature and lower BM were significant predictors of individual mortality during the Black Death.

After the Black Death, we see a significant increase in mean stature and weight in comparison to the Black Death period, and this could mean that heavier and taller individuals had more odds of surviving during the plague. Between the pre‐Black Death period and the post‐Black Death, however, we only notice a slight, not significant, increase in stature in female individuals and a decrease in males. Conversely, DeWitte and colleagues (DeWitte 2017; Brennan and DeWitte 2022) observed a little decrease in female stature after the plague pandemic, compared to the pre‐Black Death period. This can be explained by a slight difference in the sample, in particular the use by DeWitte and colleagues of the St Mary Spital infirmary cemetery for the post‐Black Death period. For this skeletal collection, the chronological information available on the WORD database did not allow us to distinguish individuals buried after 1350; therefore, they were not included in our dataset. On the other hand, the victims at St Mary Spital, a medieval hospital, might reflect the population's poorest health conditions. Moreover, it is important to note that the differences in results among the studies could be due to the different types of statistics performed. Since both stature, BM, and BMI are strongly influenced by age and sex, our adjusted multiple regression models allow a less biased picture of the changes in the three periods, as shown also by the better R2 of the adjusted models compared with the crude models.

Previous studies have noted an improvement in the health status of London's population after the plague (DeWitte 2017, 2014), which the author explain as being due to selection by the plague that killed mainly the less healthy individuals. However, after the epidemic, a general improvement in living standards in England was recorded. The severe labour shortages caused by the Black Death led to higher wages and lower prices for food, goods, and housing, as well as a decrease in social inequalities in access to food (DeWitte 2017; Dyer 2005). Improved nutritional conditions after the Black Death can be found in documentary sources that refer to lower mortality after this period (Nightingale 2005), although the documentary evidence does not cover all social classes. If plague did not select only frail individuals (Zedda et al. 2022), the general improvement in health status after the Black Death was likely due to a combination of both phenomena: a reduction of the most fragile part of the population and the general improvement in socio‐economic conditions. Improvements in stature and BM after the Black Death are slightly more pronounced among females compared to males, aligning with evidence indicating lower female mortality rates in medieval London (DeWitte 2010; Zedda et al. 2022). Sex differences in health outcomes may also reflect variations in access to healthcare. Some studies suggest a higher frequency of caries in female individuals than in males in the medieval context (Bertilsson et al. 2021; Meinl et al. 2010) as a consequence of an increase in carbohydrate consumption. Furthermore, women in medieval England prepared food and thus had preventive access to caries‐promoting foods (Walter et al. 2016). However, direct evidence of dietary differences between males and females in medieval Europe is limited (DeWitte 2017).

### 4.1 Strengths and Limitations

One of the biases of this study is the nature of the sample itself, as the Black Death sample is a catastrophic cemetery, and therefore inherently more heterogeneous in terms of both social classes and ancestry of individuals (Redfern and Hefner 2019). In contrast, the cemeteries before and after the epidemic likely had different socio‐economic compositions. Although the statistics may reveal some differences due to socio‐economic status (SES), these are minimal, as we have aimed to provide a cross‐section of the entire population. Moreover, during period C, some individuals may have died as a result of subsequent plague epidemics. Two such cases were confirmed in this study. Given that some research suggests that short stature, and by extension frailty, may have been a factor in plague mortality (e.g., DeWitte and Hughes‐Morey 2012; Zedda et al. 2022), the inclusion of individuals who died in subsequent epidemics could contribute to a homogenization of data between periods B and C. However, when the two identified individuals were excluded, the statistical analysis produced almost identical results, with no changes in significance values; therefore, they were retained.

Another limit is the availability in WORD of data on bone elements useful for defining weight status, a condition which has reduced our sample size. Furthermore, functional elements for reconstructing BM were not available for sub‐adults. The latter in particular represents a strong limitation because the scientific literature on the relationship between infectious diseases and nutrition in children is considerable and would enable interesting comparisons. Indeed, the synergistic association between malnutrition and infections shows that malnutrition (especially undernutrition) is a risk factor in children for the incidence and severity of infectious diseases (Katona and Katona‐Apte 2008; Ibrahim et al. 2017).

Additionally, in this study, the methods used to estimate BM are diverse in different individuals and employ various skeletal elements with a certain degree of error. The mechanical approach we applied has an accuracy which is acknowledged in the literature. For instance, while the morphometric approach, which relies on stature and body widths such as bi‐iliac width, is more reliable in reconstructing BM (Auerbach and Ruff 2004), the mechanical method is more easily applied because it takes into account joint size and geometric properties of the diaphyseal shaft (Lacoste Jeanson et al. 2017; Auerbach and Ruff 2004; Niskanen et al. 2018; Ruff et al. 2012, 2018). Undoubtedly, using both mechanical and morphometric methods encompassing different skeletal dimensions would have helped to better understand the variations in body size and make the estimation more accurate (Ruff et al. 2005); we would have used this approach if all measurements were available.

Stature was calculated indirectly using regression equations and, when not all bones were preserved, the available bone measurement was used for estimation.

Furthermore, in our study, we found that BMI does not provide significant insights, unlike stature and BM alone, for tracking the effects of a catastrophe such as the Black Death on a population. This suggests that, while BMI holds substantial utility in population‐level statistics to assess trends within communities, its applicability in historical contexts must be carefully evaluated (Wu et al. 2024). Such evaluation should consider not only the nature of the studies but also the influence of secular trends in body size and dietary changes over time. A future direction of this work could involve examining the association between skeletal pathologies or lesions and individuals with BMI values above or below the weight range considered normal.

This study contributes to the open debate on the identification of indicators that can provide insights into population conditions in different historical periods, contexts, and circumstances as well as the interplay between nutrition and infectious diseases (and vice versa). The general improvement of nutritional conditions in Western society, the disappearance of certain diseases, and the development of preventive and control measures have made it challenging to define comparable models for the past (Mercer 2021; Schneider 2022). Therefore, this study intends to shed new light on this issue, with the future goal of expanding the sample or comparing these data with those from similar contexts.

## 5 Conclusions

In conclusion, for the first time, BM and BMI have been used to assess the effect of a catastrophic event on a population. The results confirm that social upheavals before the Black Death generated a more frail and sick population who might have been in general more susceptible to dying from plague, as was previously theorized (DeWitte 2015, 2017; DeWitte and Hughes‐Morey 2012; DeWitte and Wood 2008); while after the extreme devastation of the Black Death, the population was in better health conditions, as evidenced by improvements in stature and BM.

## Author Contributions

**Jessica Mongillo:** conceptualization (equal), data curation (lead), formal analysis (equal), investigation (equal), methodology (lead), supervision (equal), validation (equal), visualization (equal), writing – original draft (equal), writing – review and editing (equal). **Anthea Cerviero:** conceptualization (equal), data curation (lead), methodology (lead), validation (equal), writing – original draft (equal), writing – review and editing (equal). **Nicoletta Zedda:** conceptualization (supporting), data curation (equal), investigation (equal), methodology (supporting), supervision (equal), writing – original draft (equal), writing – review and editing (equal). **Natascia Rinaldo:** conceptualization (lead), data curation (equal), formal analysis (equal), investigation (equal), methodology (supporting), supervision (lead), validation (equal), writing – original draft (equal), writing – review and editing (equal). **Barbara Bramanti:** conceptualization (lead), data curation (equal), formal analysis (equal), investigation (equal), methodology (equal), supervision (lead), validation (equal), writing – original draft (equal), writing – review and editing (equal).

## Conflicts of Interest

The authors declare no conflicts of interest.

## Supporting information


**Table S1.** Mean stature, BM and BMI in the sample divided by sex and age class.

## Data Availability

The data on the osteological collections is freely accessible for research purposes in the Wellcome Osteological Research Database (WORD) by the Museum of London under request at https://www.museumoflondon.org.uk/collections/other‐collection‐databases‐and‐libraries/centre‐human‐bioarchaeology/osteological‐database.
