# Supplementary material for: A Heavy Issue: Changes in Body Size in London Before, During and After the Black Death
Source: Am J Biol Anthropol. 2025 Jul 17;187(3):e70098. doi: 10.1002/ajpa.70098 (PMC12272036; doi:10.1002/ajpa.70098)
Supplement: Supplementary file 1 — Table S1. Mean stature, BM and BMI in the sample divided by sex and age class. [file AJPA-187-e70098-s001.docx]

Table S1: mean stature, BM and BMI in the sample divided by sex and age class

|  | **M** | | | **F** | | |
| --- | --- | --- | --- | --- | --- | --- |
| **Age** | **YA** | **MA** | **OA** | **YA** | **MA** | **OA** |
| A | | | | | | |
| N | **31** | **40** | **9** | **12** | **12** | **11** |
| Stature (cm)  Mean (SD) | 172.2 (4.9) | 171.6 (7.1) | 173.1 (5.0) | 158.5 (3.7) | 159.4 (5.4) | 159.1 (6.9) |
| BM (kg)  **Mean (SD)** | 76.2 (10.1) | 76.3 (8.1) | 78.5 (3.4) | 63.9 (10.2) | 60.4 (7.8) | 61.9 (5.2) |
| BMI (kg/m2)  **Mean (SD)** | 25.3 (2.2) | 26.0 (2.9) | 26.4 (1.5) | 24.9 (3.3) | 24.3 (3.49) | 24.5 (1.8) |
| **B** | | | | | | |
| **N** | 69 | 48 | 10 | 34 | 19 | 4 |
| Stature (cm)  **Mean (SD)** | 169.4 (5.4) | 168.4 (4.5) | 166.3 (4.5) | 158.2 (5.5) | 157.0 (4.6) | 154.6 (2.7) |
| BM (kg)  **Mean (SD)** | 72.3(6.9) | 73.1 (7.2) | 76.7 (5.7) | 60.6 (6.6) | 50.9 (7.4) | 60.9 (6.8) |
| BMI (kg/m2)  **Mean (SD)** | 25.4(2.3) | 26.4 (2.7) | 27.8 (1.8) | 24.3 (2.3) | 24.3 (3.3) | 24.4 (2.4) |
| C | | | | | | |
| **N** | 28 | 37 | 14 | 15 | 13 | 2 |
| Stature (cm)  **Mean (SD)** | 169.5 (7.8) | 171.1(4.9) | 170.1 (5.0) | 159.2 (6.1) | 162.4 (5.1) | 161.2 (4.7) |
| BM (kg)  **Mean (SD)** | 73.9 (6.4) | 73.8 (7.6) | 77.8 (5.3) | 60.9 (5.6) | 64.1 (8.8) | 57.7(0.5) |
| BMI (kg/m2)  **Mean (SD)** | 25.7 (1.9) | 25.3 (2.0) | 27.6(2.3) | 24.6(2.2) | 24.2 (2.1) | 22.2 (1.5) |

**M: males; F: females; YA: 18 – 35years of age; MA: 36 – 45 years of age: OA: > 46 years of age;**
